# Root tolerance and biochemical response of Chinese lettuce (*Lactuca sativa* L.) genotypes to cadmium stress

**DOI:** 10.7717/peerj.7530

**Published:** 2019-08-22

**Authors:** Mohammed Mujitaba Dawuda, Weibiao Liao, Linli Hu, Jihua Yu, Jianming Xie, Alejandro Calderón-Urrea, Xin Jin, Yue Wu

**Affiliations:** 1 Department of Horticulture, Faculty of Agriculture, University for Development Studies, Tamale, Ghana; 2 College of Horticulture, Gansu Agricultural University, Lanzhou, China; 3 Department of Biology, California State University, Fresno, Fresno, CA, United States of America; 4 College of Agriculture and Forest Science, Linyi University, Linyi, Shandong Province, China

**Keywords:** Cadmium stress, Antioxidant enzymes, Endogenous hormones, Lettuce genotypes, Root morphology

## Abstract

This study was conducted to determine the root tolerance and biochemical responses of four Chinese *Lactuca sativa* L. genotypes (*Lüsu, Lümeng, Yidali* and *Anyan*) to cadmium (Cd^2+^) stress. Twenty-eight days old seedlings were exposed to Hoagland’s nutrient solution supplied with or without 100 µM CdCl_2_ and monitored for seven days in a climate controlled room. The 100 µM CdCl_2_ significantly (*P* < 0.001) decreased all the root morphological indexes of the four genotypes. However, *Yidali*, which possessed the smallest root system, exhibited greater root tolerance to Cd^2+^ by having the highest tolerance indexes for root volume (46%), surface area (61%), projected area (74%) and numbers of root forks (63%) and root tips (58%). Moreover, Cd^2+^ stress also caused increases in H_2_O_2_ contents in the roots but the increase was least in *Yidali* which showed greater root tolerance to Cd^2+^stress. The effect of Cd^2+^ stress on the contents of hormones in the roots depended on the genotypes. Under Cd^2+^ stress, abscisic acid correlated positively with indole-3-acetic acid (*r* = 0.669*), gibberellic acid (*r* = 0.630*) and cytokinin (*r* = 0.785**). The antioxidant enzyme activities and proline responses of the four genotypes to Cd^2+^ stress were similar. The SOD activity was decreased whiles the CAT and POD activities, as well as the contents of proline increased in all the genotypes under the stress condition. These results suggest that lettuce genotypes with smaller root systems could be more tolerant to Cd^2+^ stress compared to those with larger root systems.

## Introduction

Heavy metals are a major cause of abiotic stress in plants and their accumulation in human and animal tissues leads to serious health problems (Singh et al., in press). The oxidative stress caused by heavy metals including cadmium (Cd^2+^) results in membrane damage and also decreases photosynthesis (Su et al., 2017; Kaur et al., 2017). Increases in the levels of reactive oxygen species (ROS) such as hydrogen peroxide (H_2_O_2_), superoxide anion and thiobarbituric acid reactive substances (TBARS) in plant cells is the main cause of oxidative stress in plants (Tripathi et al., 2017). Cd^2+^ toxicity increases the accumulation of H_2_O_2_, superoxide anion and TBARS in lettuce (Wang et al., 2019) and in *Arabidopsis thaliana* (Song et al., 2017). The effect of Cd^2+^ on root morphology varies with different plant species (Li et al., 2009; Lu et al., 2013). Lambrechts et al. (2013) found that the root diameter of *Lolium perenne* and *Trifolium repens* increases in relation to a high number of short and wide adventitious roots under Cd^2+^ stress. Moreover, in *Zea mays* (Maksimovi’c et al., 2007) and in *Salix alba* (Lunáčková et al., 2004) plants, the root diameters increases under Cd^2+^ stress. The root length of lettuce (*Lactuca sativa* L.) plants exposed to Cd^2+^ decreased by 89% (Bautista, Fischer & Cárdenas, 2013). In *A. thaliana*, Cd^2+^ stress decreased root length but doubled the diameter of the lateral roots (Vitti et al., 2013). In view of the variations in the response of plants root systems to Cd^2+^ stress, Lu et al. (2013) indicated that the response of plants root systems to Cd^2+^ stress can be used in assessing their tolerance to Cd^2+^ stress.

Plant hormones are a group of chemical messengers that are primarily known to regulate plant growth and development (Peto’ et al., 2011) but they also play roles in plants tolerance to biotic and abiotic stresses (Krishnamurthy & Rathinasabapathi, 2013). Christmann et al. (2006) reported that increases in endogenous abscisic acid (ABA) contents in plants improve their tolerance to various stresses. The endogenous levels of ABA in rice (Kim et al., 2014) and potato tubers (Stroinski et al., 2010) as well as in the roots of two aquatic plants, *Typha latifolia* and *Phragmites australis* (Fediuc, Lips & Erdei, 2005) increased under Cd^2+^ stress. Veselov et al. (2003) also found that the contents of cytokinin in the shoots and roots of wheat decreased within 1–2 h of exposure to Cd^2+^. Auxins, including indole-3-acetic acid (IAA) also play important roles in protecting and regulating plants metabolism under stress conditions. Generally, heavy metal stress decreases the levels of endogenous auxins (Gangwar et al., 2014).

Plants tolerance to heavy metals has been associated with increase activities of antioxidant enzymes (Yang & Ye, 2015). However, Fodor (2002) reported that high Cd^2+^ concentrations resulted in decreased antioxidant activities in plants. The catalase (CAT) activity in several plants including, *Lactuca sativa* (Wang et al., 2019; Monteiro et al., 2009), *Phaseolus vulgaris* (Chaoui et al., 1997) and *Pisum sativum* (Chaoui & El Ferjani, 2005) decreased when the plants were subjected to Cd^2+^ stress. The CAT activity in pea seedlings was also decreased whiles the superoxide dismutase (SOD) and peroxidase (POD) activities increased under Cd^2+^ stress (Agrawal & Mishra, 2007). The rates of POD activity in the roots and shoots of pea seedlings were higher at lower Cd^2+^ (5–20 µM CdCl_2_) concentrations than at higher Cd^2+^ (50–100 µM CdCl_2_) concentrations (Bavi, Kholdebarin & Morashahi, 2011). Proline, which is a component of the non-specific defense systems against heavy metal toxicity acts as a chelator as well as a protein stabilizer in plants (Sharma & Dubey, 2005). The contents of proline increased in lettuce plants under Cd^2+^ stress (Jibril et al., 2017) and in wheat plants exposed to Pb stress (Lamhamdi et al., 2013).

Different plant species possess a wide range of protection mechanisms against Cd^2+^-induced stress (Mohamed et al., 2012). However, the response of plants to Cd^2+^ stress depend on several factors including the genotype and the Cd^2+^ concentration of the growing medium (Irfan, Ahmad & Hayat, 2013). The root system of plants is the foremost organ that comes into contact with Cd^2+^ in a growing medium. Therefore, the response of the root system to Cd^2+^stress could have greater impact on the tolerance of the whole plant to the stress. Richard et al. (2015) screened maize lines for Al tolerance and indicated that root tolerance should be considered in selecting cultivars for Al tolerance. In *Brassica juncea* seedlings exposed to Cd^2+^ stress, the POD and CAT activities of the root system were more efficient in scavenging ROS compared with the shoot system (Mohamed et al., 2012). Although a number of studies have reported the effect of Cd^2+^ on lettuce plants (Wang et al., 2019; Bautista, Fischer & Cárdenas, 2013; Monteiro et al., 2009; Monteiro et al., 2007), the root tolerance and biochemical response of the root system of different lettuce genotypes to Cd^2+^ stress have rarely been reported. Moreover, to the best of our knowledge, the response of the genotypes studied in the present experiment to Cd^2+^ stress has not been reported.

The objectives of our study were: (1) to evaluate the root tolerance of four lettuce genotypes to Cd^2+^ stress based on root tolerance indexes, (2) to determine the biochemical (hormones, antioxidant enzymes activities and proline) defense response of the four lettuce genotypes to Cd^2+^ stress.

## Materials and Methods

### Genotypes used for the experiment

The experiment was conducted at the College of Horticulture, Gansu Agricultural University, Lanzhou, Gansu Province, PR China (36°03′N, 103°40′E). The seeds of four improved lettuce (*Lactuca sativa* L.) genotypes obtained from the Gansu Academy of Agricultural Sciences, Lanzhou, China, were used in this experiment (Table 1).

### Seedling growth conditions

The seeds were germinated in petri dishes within 48 h under light condition in a climate box (temperature 20 °C; light intensity 200 µmol m^−2^ s^−1^ photosynthetically active radiations, *PAR*; relative humidity 80%). The germinated seeds were sown in trays which were filled with vermiculite and the seedlings were raised in a greenhouse (average daily temperature 24 ± 2 °C; relative humidity 60–70%; 12 h light) for two weeks. During the first two weeks of growth, the young seedlings were irrigated with half-strength Hoagland’s nutrient solution with pH adjusted to 6.0 (Hoagland & Arnon, 1950). After the first two weeks of growth, the seedlings were grown hydroponically for another two weeks using full-strength Hoagland’s nutrient solution in a climate controlled room (25 °C/22 °C light/dark periods, 75% relative humidity and 14 h light period with 200 µmol m^−2^ s^−1^ *PAR*). In all, 240 seedlings were transferred to 24 opaque hydroponic containers (length 82 cm; width 30 cm; height 5 cm). Each of the containers were filled with 5 L of the full-strength Hoagland’s nutrient solution and supplied with 10 seedlings of a particular lettuce genotype. The nutrient solution was changed at 4 days interval until the seedlings were four weeks old before the 100 µM CdCl_2_ treatment was applied according to the experimental design.

**Table 1:** Lettuce genotypes used for the study.

Genotype	Days to maturity from transplanting	Estimated yield (kg/ha)	Special attributes
*Yidali*	40	22,500	Resistant to low temperature and moisture stress, early maturing
*Lümeng*	60	22,500	Resistant to diseases and low temperature
*Lüsu*	–	–	High yielding
*Anyan*	40–50	30,000–45,000	Resistant to diseases, low temperature and moderate heat stress

#### Experimental design and treatments

A 4 × 2 factorial experiment in a completely randomized design with 3 replications was conducted in the climate controlled room using 28 days old seedlings. The treatments consisted of the four lettuce genotypes and two Hoagland’s nutrient solutions. Two hundred and forty seedlings (60 seedlings per genotype) were grouped into two. Half of the seedlings of each genotype were exposed to the Hoagland’s nutrient solution supplemented with 100 µM CdCl_2_ and the other half were maintained in the nutrient solution without 100 µM CdCl_2_, as control plants. We used the 100 µM CdCl_2_ concentration in our experiment based on previous studies that were conducted with lettuce as the test crop (Xu et al., 2014; Monteiro et al., 2009; Monteiro et al., 2007). At seven days after exposure to treatments, one plant each was taken randomly from each experimental unit and used for the analysis of root morphological indexes. At the same time, five plants were also taken randomly from each experimental unit and their root tissues were immediately stored at −80 °C for biochemical analysis.

### Data measurements

#### Root morphological indexes

The roots of the sampled plants were gently separated from the shoots, rinsed in distilled water and the root systems were scanned with the aid of a root scanner (STD 4800, EPSON, Canada) to obtain the digital images. The total root length (TRL), root volume (RV), root surface area (RSA), root projected area (RPA), and numbers of root tips (NRT) and root forks (NRF) per plant were determined using the root image analysis software, Win RHIZO version 5.0 (Regent Instruments, Inc., Quebec City, Canada).

#### Determination of root tolerance indexes

The Cd^2+^ tolerance indexes (TI) of the plants root systems which indicates the resistance of the root systems to the heavy metal stress was calculated as described by Wilkins (1978) with some modifications. Since the various root morphological indexes such as total root length, root volume, number of root tips and root surface area have direct influence on Cd^2+^ uptake and the plants tolerance to Cd^2+^ stress, we calculated the Cd^2+^ TI for each of the root indexes at the end of the experiment. Based on the TI score, we considered the genotype which had the highest TI for most of the indexes measured as the tolerant genotype among the genotypes we evaluated. The TIs were calculated as follows:

\begin{eqnarray*}\mathrm{TI}= \frac{\text{Root index under Cd stress}}{\text{Root index without Cd stress}} \times 100. \end{eqnarray*}

#### Determination of H_2_O_2_ contents in root samples

Contents of H_2_O_2_ in the root samples were determined following the procedure described by Junglee et al. (2014) but with slight modifications. Briefly, we 0.1 g fresh root sample was ground with liquid nitrogen in mortar with pestle and the homogenate transferred into a 2 mL capacity centrifuge tube which was then kept in ice bath. We added 1.5 mL 0.1% TCA and the homogenate were centrifuged at 12,000× g for 15 min under 4 °C. Then, 0.5 ml of the supernatant was thoroughly mixed with 0.5 mL PBS (pH 7.0) and 1 mL 1 mol L^−1^ KI. The mixture was placed under constant temperature (28 °C) for 1 h. The absorbance was measured at 390 nm and the contents of H_2_O_2_ were calculated using the H_2_O_2_ reference standard curve (0, 1, 2, 3, 4 and 5 mmol L^−1^).

#### Determination of contents of hormones in root samples

Fresh root samples (0.5 g) of each of the four lettuce genotypes were collected from three plants in each experimental unit, quickly wrapped in alumnum foil, labeled and then put in liquid nitrogen. After this, the samples were put in labeled plastic bags and stored in a freezer at −80 °C. The contents of ABA, IAA, GA_3_ and Cytokinin were later determined by the Enzyme Linked Immunosorbent Assay (ELISA) technique (Shanghai Jiwei Biological Technology Co. Ltd., PR China).

#### Antioxidant enzymes activities and proline contents in roots

Enzymes assays were done using frozen root tissues that were stored at −80 °C. For each enzyme, about 0.5 g of the root tissue was ground in liquid nitrogen with a mortar and pestle and then homogenized in 5 ml of 0.1 M phosphate buffer (pH 7.5), containing 0.5 mM ethylene-diamine-tetra-acetic acid (EDTA). Each homogenate was centrifuged at 12,000× g for 15 min at 4 °C, and the supernatant was aliquoted for determination of enzymatic activity; superoxide dismutase (SOD, EC 1.15.1.1), catalase (CAT, EC 1.11.1.6) and peroxidase (POD, EC 1.11.1.7) activity assays. The SOD activity was measured by determining the ability of plants to inhibit the photochemical reduction of nitroblue tetrazolium (NBT). Based on the inhibition of 50% of NBT photoreduction as one unit of SOD activity, the amount of reduced NBT was monitored spectrophotometry–colorimetry at 560 nm (Giannopolitis & Ries, 1977). The POD activity was estimated at 470 nm as described by Chance & Maehly (1955). The CAT activity was measured by determining the oxidation of H_2_O_2_ and monitored as a decline spectrophotometry-colorimetry at 240 nm (Nakano & Asada, 1981). Proline contents in the root samples were determined after extraction at room temperature with 3% of 5-sulfosalicylic acid solution as previously described (Bates, Waldren & Teare, 1973).

### Data analysis

The data were subjected to analysis of variance and the treatment means were separated by the least significance difference (LSD) test at 5% using Genstat statistical software (9th edition; Lawes Agricultural Trust, Rothamsted Experimental Station, UK). In the figures, the spread of the mean values were shown using the standard deviations. Different letters assigned to the bars indicate significant differences among the treatments by LSD (*P* < 0.05). Pearson’s correlation analysis was used to determine the relationship between root morphological indexes and contents of root hormones under no stress and under Cd^2+^ stress conditions. The correlation analyses were done using the average values for every trait × genotype × treatment. Graphs were prepared using Graphpad Prism version 8.0.

## Results

### Root morphological characteristics

**Figure 1:**
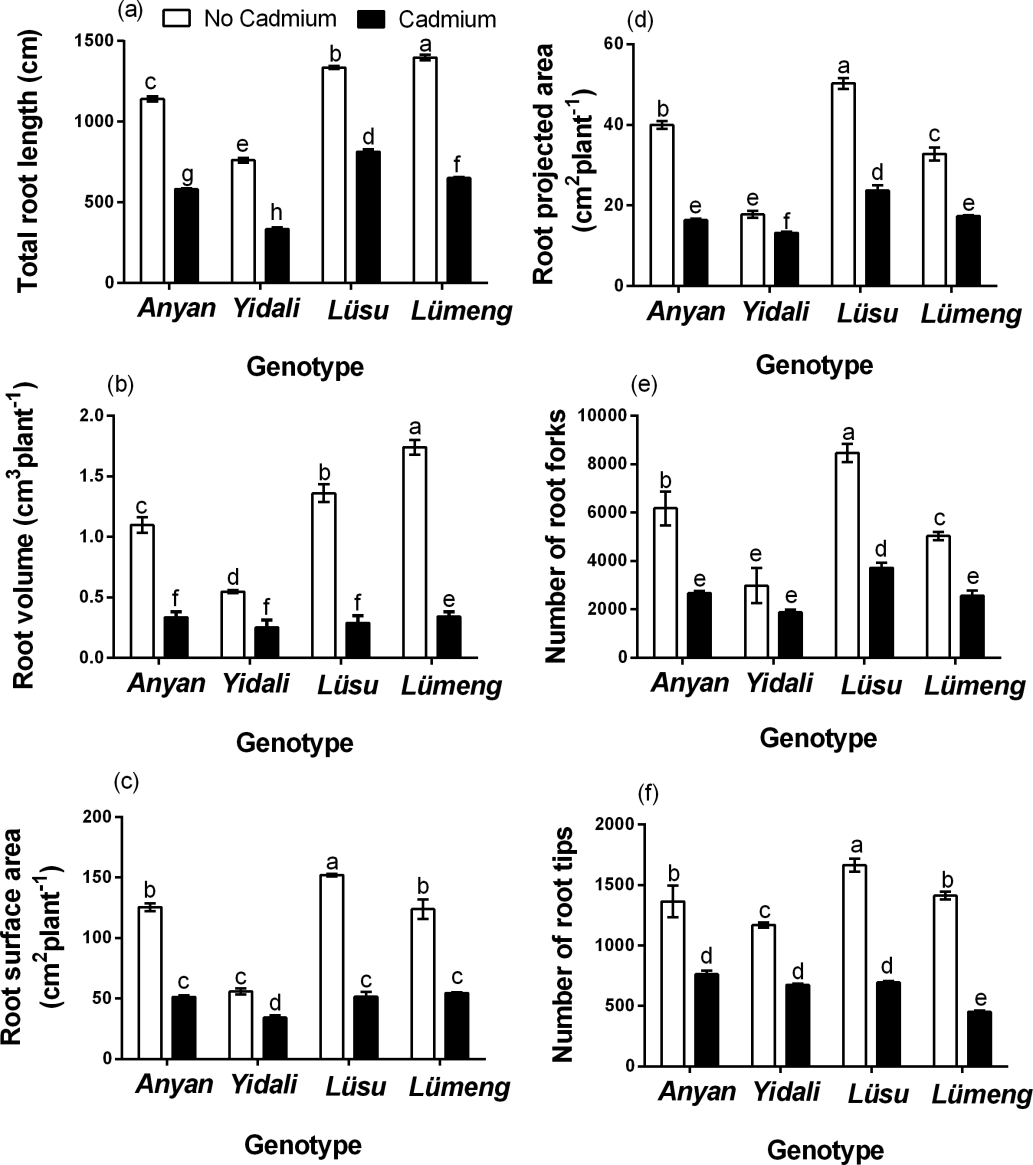
Effectof 100 µM CdCl_2_ on (A) TRL (B) RV (C) RSA (D) RPA (E) NRF and (F) NRT of our lettuce genotypes at seven days after treatments.

**Figure 2:**
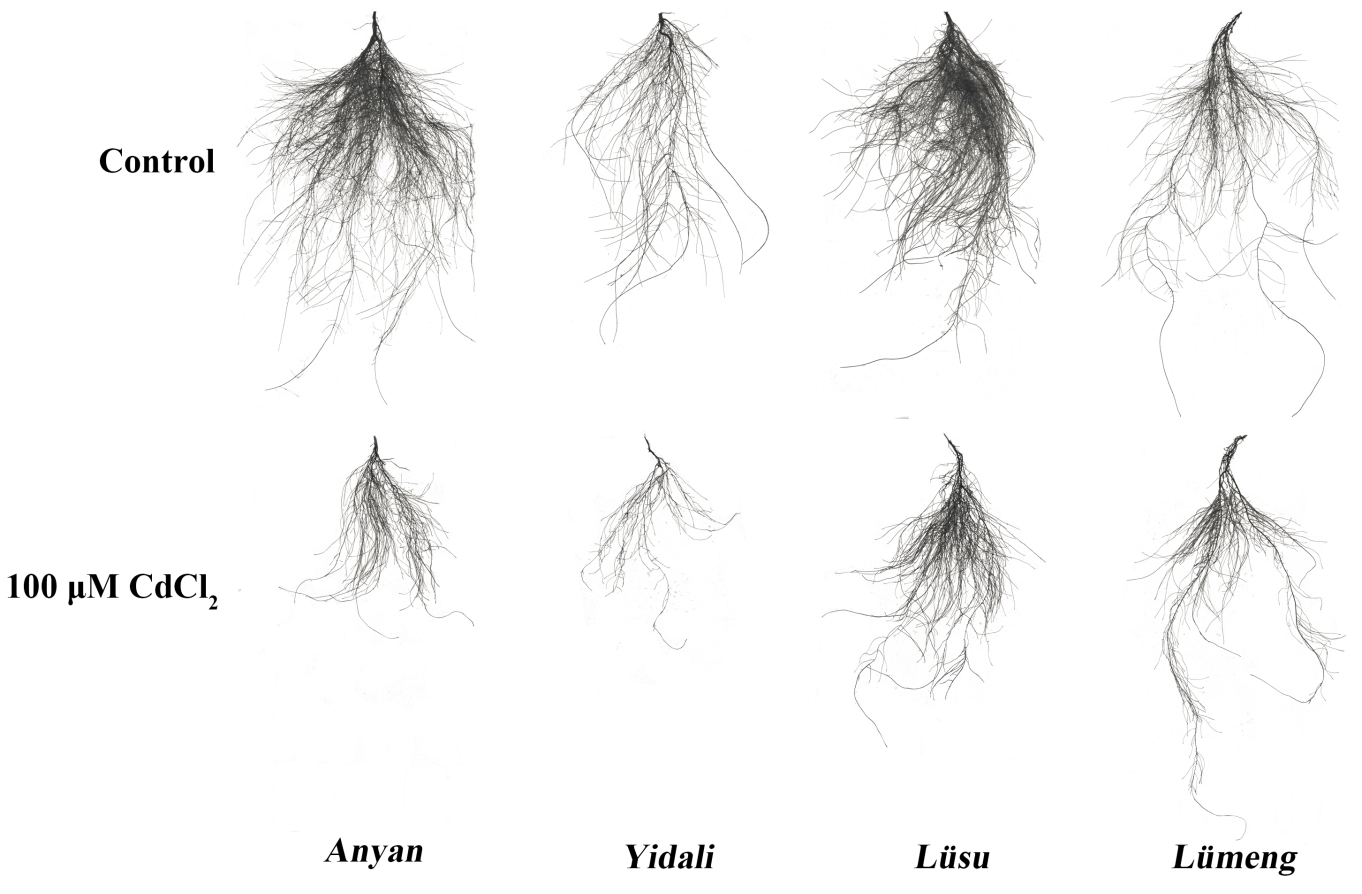
Scan images of the root systems of four lettuce genotypes at seven days after 100 µM CdCl_2_ treatment.

The results of our experiment showed that there was significant (*P* < 0.001) genotype × Cd^2+^ interaction effect on total root length (TRL), root volume (RV), root surface area (RSA), root projected area (RPA), number of root forks (NRF) and number of root tips (NRT) of the lettuce genotypes (Fig. 1). Generally, Cd^2+^ stress decreased all the root morphological indexes across the genotypes but *Yidali* was the least affected. The decrease in TRL due to Cd^2+^ was least (39.0%) in *Lüsu* and greatest (56.0%) in *Yidali*. The least (38.7%) decrease in RSA was observed in *Yidali* and the greatest (65.9%) in *Lüsu*. Moreover, the least (25.6%) and the greatest (59.1%) decreases in RPA were observed in *Yidali* and *Anyan* genotypes, respectively. The least (54.0%) decrease in RV also occurred in *Yidali* whiles the greatest (80.3%) was observed in *Lümeng*. Cd^2+^ stress also decreased NRT and NRF by 42.3%–67.9% and 36.0%–56.8%, respectively in the genotypes and in each case, *Yidali* was the least affected. The decreases in the root morphological indexes due to Cd^2+^ stress resulted in reductions in the sizes of the root systems of the four lettuce genotypes (Fig. 2).

### Root tolerance to cadmium stress

**Figure 3:**
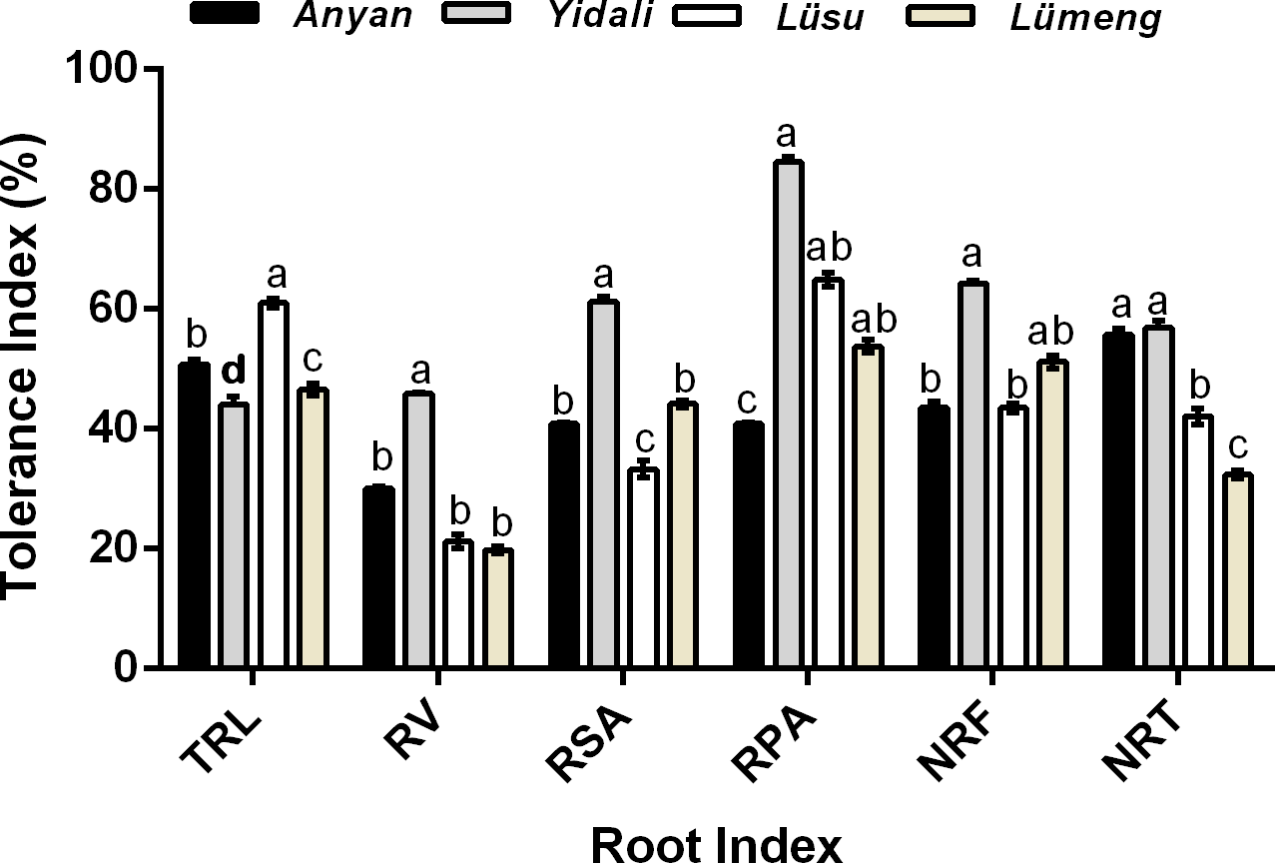
Root Cd^2+^ tolerance indexes(%) of four lettuce genotypes after 100 µM CdCl_2_ treatment.

Figure 3 shows the results of the root tolerance of the four genotypes to Cd^2+^ stress. There was significant (*P* < 0.001) variations in the tolerance indexes (TIs) among the four lettuce genotypes. The result showed that at seven days after plants exposure to Cd^2+^, *Yidali*, had the highest TI for most of the indexes measured including RV (46%), RSA (61%), RPA (84%), NRF (65%) and NRT (58%). Based on this result, we considered the *Yidali* genotype as the most tolerant genotype among the genotypes evaluated. With the exception of TI for TRL which was highest (61%) in *Lüsu ,* the other genotypes had lower TI scores compared with *Yidali* and these genotypes were considered as sensitive to Cd^2+^ stress.

#### Contents of H_2_O_2_ in roots

**Figure 4:**
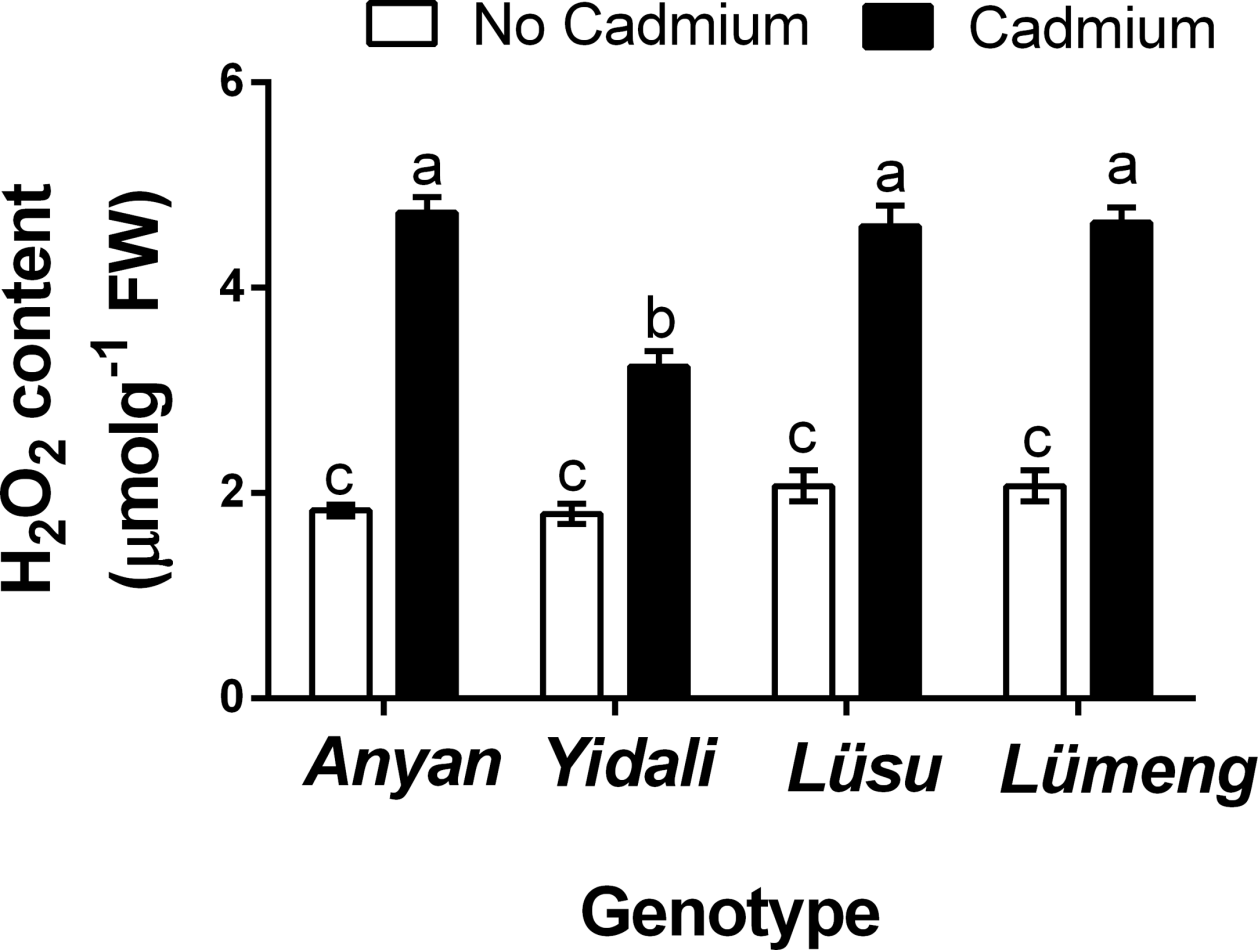
Effect of 100 µM CdCl_2_ on H_2_ O_2_ contents in the roots of four lettuce genotypes after treatments.

The results showed significant (*P* < 0.001) genotype × Cd^2+^ interaction effect on contents of H_2_O_2_ in the roots of the lettuce genotypes (Fig. 4). Generally, H_2_O_2_ contents increased in all the genotypes under Cd^2+^ as compared to their respective control plants. In comparison with control plants, Cd^2+^ increased H_2_O_2_ contents in the roots of the four genotypes by 44.3–61.1%. The highest H_2_O_2_ content was found in *Anyan* but this was statistically similar to the H_2_O_2_ contents in the *Lüsu* and *Lümeng* genotypes. The lowest H_2_O_2_ content was found in the *Yidali* plants which were exposed to Cd^2+^ treatment.

#### Contents of root hormones

**Figure 5:**
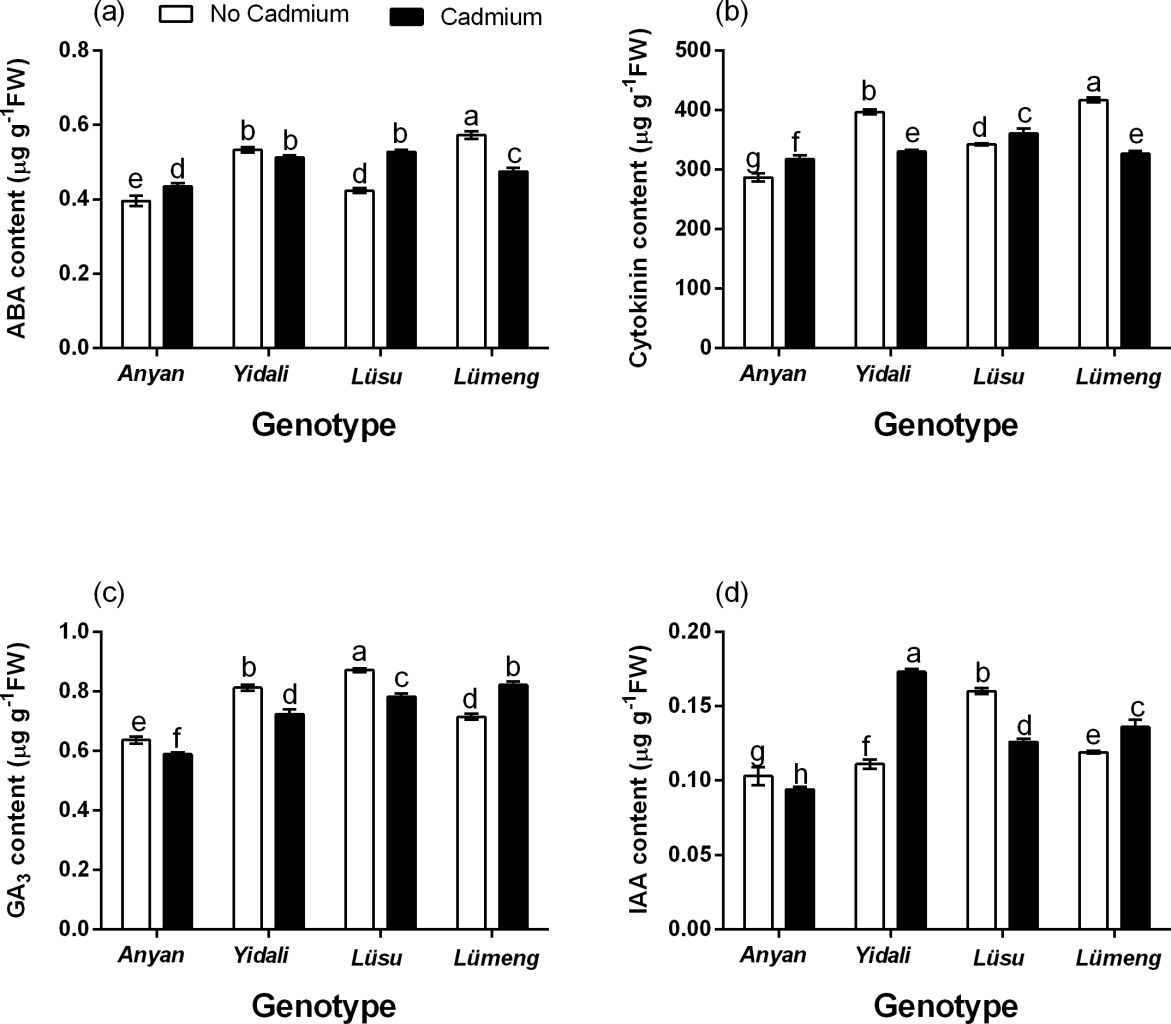
Effect of 100 µM CdCl_2_ on hormones contents in the roots of four lettuce genotypes after treatments. (A) Shows the changes in ABA contents in the roots. (B) Shows the changes in cytokinin contents in the roots. (C) Shows the changes in GA_3_ contents in the roots and (D) Shows the changes in IAA contents in the roots. Data shown are treatment means ± SD of 3 replications. Bars assigned with different lower case letters indicate significant differences by LSD test (*P* < 0.05). ABA, Abscisic acid; GA_3_, Gibberellic acid and IAA, Indole-3-acetic acid.

The results showed significant (*P* < 0.001) genotype × Cd^2+^ interaction effect on contents of abscisic acid (ABA), cytokinin, gibberellic acid (GA_3_) and indole-3-acitic acid (IAA) in the roots of the four lettuce genotypes (Fig. 5). The contents of ABA in *Lüsu* and *Anyan* plants under Cd^2+^ treatment were 19.8% and 8.9% more than their respective control plants. However, the ABA contents in *Lümeng* and *Yidali* genotypes were 17.1% and 3.9% lower compared with the control. The contents of GA_3_ in *Anyan, Yidali* and *Lüsu* plants under Cd^2+^ stress were 7.3%, 10.9% and 10.3% respectively lower than their control plants. However, the content of GA_3_ in *Lümeng* under the stress condition increased by 12.9% compared with the control plants. The contents of cytokinin in *Anyan* and *Lüsu* increased by 9.7% and 5.1% but decreased by 21.6% and 16.8% in *Lümeng* and *Yidali* under Cd^2+^ stress. The highest IAA content was found in the *Yidali* genotype under Cd^2+^ treatment and this was 36.2% more than the control. However, the IAA contents in *Lüsu* and *Anyan*, under Cd^2+^ treatment were 21.6% and 8.7%, lower than their respective control plants.

### Correlation analysis

Table 2 shows the correlation analyses for root morphological indexes and root hormones of the four lettuce genotypes under Cd^2+^-free condition (diagonal, white background) and under Cd^2+^ stress (diagonal, grey background). Under no Cd^2+^ stress, the correlations among most of the root indexes were significantly positive. For instance, total root length (TRL) correlated positively with root surface area (RSA) (*r* = 0.903**), root projected area (RPA) (*r* = 0.756**), number of root forks (NRF) (*r* = 0.699*), number of root tips (NRT) (*r* = 0.774**) and root volume (RV) (*r* = 0.960**). Moreover, IAA correlated positively with RSA (*r* = 0.590*), NRF (*r* = 0.698*), NRT (*r* = 0.799**) and RPA (*r* = 0.657*). However, the correlation between cytokinin and RPA was negative (*r* =  − 0.579*). The correlation between GA_3_ and IAA was positive (*r* = 0.765**) whiles that between ABA and cytokinin was also positive (*r* = 0.764**) under Cd^2+^-free condition. The results also showed positive and negative correlations between the hormones and root morphological indexes under Cd^2+^ stress condition (Table 2, above diagonal, grey background). Total root length (TRL) correlated positively with RSA (*r* = 0.829**), NRF (*r* = 0.925**) and RPA (*r* = 0.963**). Moreover, GA_3_ correlated negatively (*r* =  − 0.768**) with NRT and IAA also correlated negatively (*r* =  − 0.732**) with RSA. However, among the hormones, only cytokinin correlated positive with TRL (*r* = 0.578*), NRF (*r* = 0.703*) and RPA (*r* = 0.791**) under the Cd^2+^ stress condition. The correlation analysis among the hormones showed that ABA correlated positively with GA_3_ (*r* = 0.630*), cytokinin (0.786**) and IAA (*r* = 0.669*) under Cd^2+^ stress.

**Table 2:** Pearson’s correlations matrix describing the relationship between the root morphological characteristics and root hormones in lettuce plants under no cadmium stress (diagonal with white background) and under cadmium stress (diagonal with grey background) at seven days after treatments.

**Index**		**1**	**2**	**3**	**4**	**5**	**6**	**7**	**8**	**9**	**10**
1.	TRL		0.829^**^	0.925^**^	−0.117	0.936^**^	0.302	0.095	0.338	0.578^*^	−0.571
2.	RSA	0.903^**^		0.660^*^	−0.268	0.592^*^	0.659^*^	−0.38	0.153	0.064	−0.732^**^
3.	NRF	0.699^*^	0.893^**^		0.159	0.954^**^	0.222	0.258	0.209	0.703^*^	−0.508
4.	NRT	0.774^**^	0.878^**^	0.879^**^		0.037	−0.215	−0.061	0-.768^**^	0.075	−0.331
5.	RPA	0.756^**^	0.950^**^	0.963^**^	0.886^**^		0.051	0.364	0.365	0.791^**^	−0.392
6.	RV	0.960^**^	.769^**^	0.507	.624^*^	.587^*^		−0.48	−0.068	−0.342	−0.457
7.	ABA	0.139	−0.204	−0.444	−0.232	−0.38	0.319		0.630^*^	0.785^**^	0.669^*^
8.	GA_3_	−0.106	−0.053	0.182	0.302	0.068	−0.173	0.069		0.511	0.511
9.	Cyto	−0.057	−0.424	−0.54	−0.241	-.579^*^	0.130	.764^**^	0.348		0.123
10.	IAA	0.492	0.590^*^	0.698^*^	0.799^**^	0.657^*^	0.372	−0.033	0.765^**^	0.033	

**Notes.**

** Correlation is significant at the 0.01 level (2-tailed).

* Correlation is significant at the 0.05 level (2-tailed). The correlation analysis were done using the three values (three replications) for every trait × cultivar × treatment.

TRL total root length RSA root surface area NRF number of root forks NRT number of root tips RPA root projected area RV Root volume ABA abscisic acid GA3 gibberellic acid Cyto cytokinin IAA indole-3-acitic acid

### Antioxidant enzymes activities and proline contents

**Figure 6:**
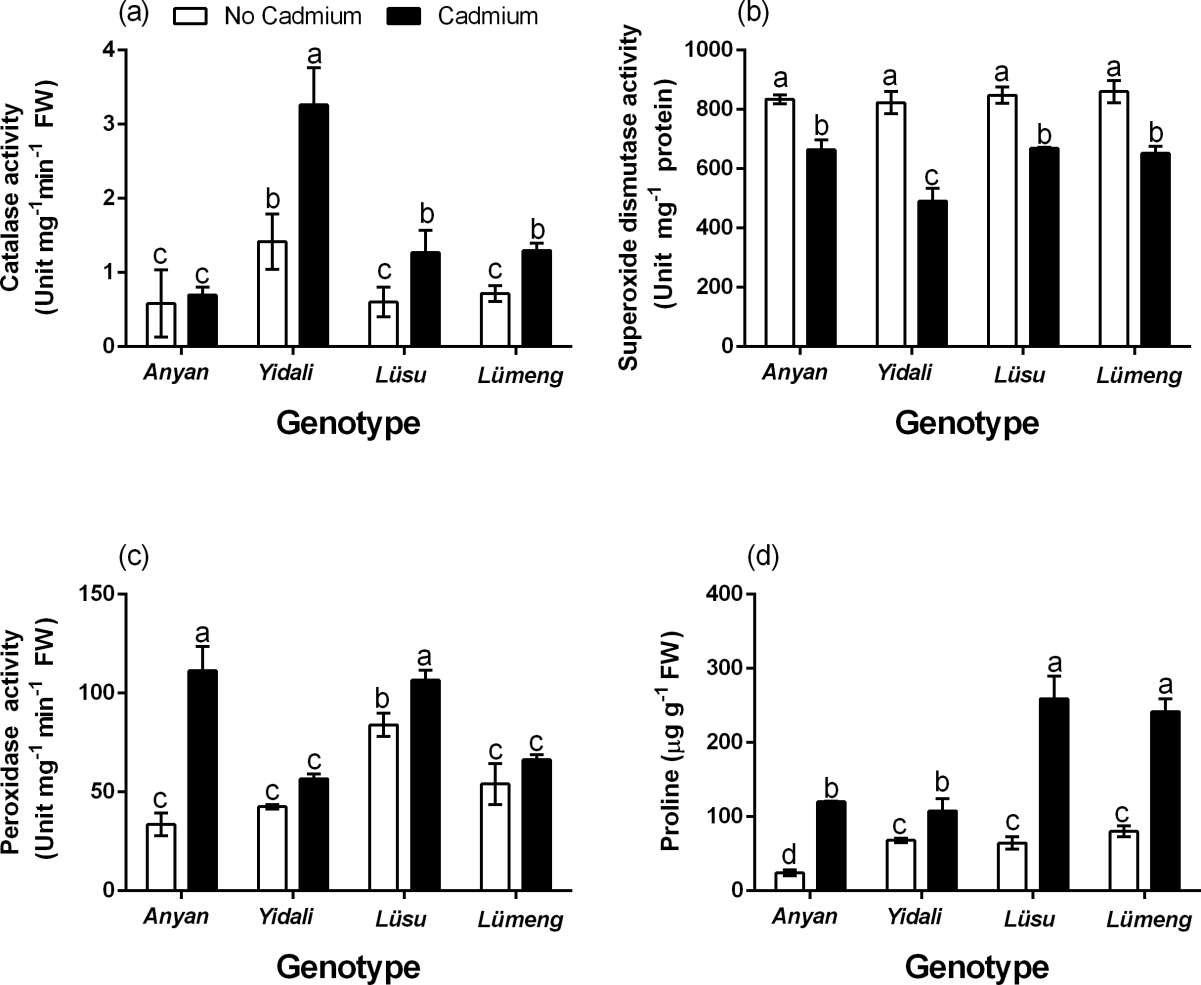
Effect of 100 µM CdCl_2_ on enzyme activities and proline. (A) Shows the activities of catalase in the roots. (B) Shows the activities of superoxide dismutase in the roots. (C) Shows the activities of peroxidase in the roots and (D) proline contents. Data shown are treatment means ± SD of 3 replications. Bars assigned with different lower case letters indicate significant differences by LSD test (*P* < 0.05).

The results also showed significant (*P* < 0.001) genotype × Cd^2+^ interaction effect on CAT, SOD and POD activities and contents of proline in the roots of the four lettuce genotypes (Fig. 6). In comparison with their respective control plants, Cd^2+^ stress decreased the SOD activities in the roots of all the genotypes by 20.4–40.3%. However, the CAT and POD activities as well as the contents of proline in all the genotypes increased by 16.7–56.6%, 18.6–69.8% and 36.7–79.8%, respectively. The greatest increase in CAT activity was observed in the *Yidali* genotype whiles the *Anyan* genotype had the highest POD activity and proline content.

## Discussion

Cadmium (Cd^2+^) interferes with the uptake of water and nutrients and causes injuries to plant roots (Jibril et al., 2017). Wang et al. (2015) reported that Cd^2+^ stress decreases total root length (TRL), root surface area (RSA) and root volume (RV) in soybean cultivars. The decrease in root length of lettuce plants due to Cd^2+^ stress was as high as 89% (Bautista, Fischer & Cárdenas, 2013). The results of our experiment also showed that the addition of 100 µM CdCl_2_ to the nutrient solution decreased the TRL, RSA, RV and root projected area (RPA) of the lettuce genotypes. The numbers of root tips (NRT) and root forks (NRF) in the studied lettuce genotypes were also decreased. This resulted in a decrease in the size of the root systems in all four genotypes. The negative correlations observed among some of the root morphological indexes under Cd^2+^ stress was indicative of the adverse effect of Cd^2+^ on the morphological indexes of lettuce roots. The tolerance of plants to Cd^2+^ stress can be influenced by the characteristics of the roots. Wang et al. (2015) reported that soybean cultivars with larger root systems were tolerant to Cd^2+^ whiles those with smaller root systems were sensitive. However, the results of our experiment showed that *Yidali*, which possessed the smallest root system, had the greatest root tolerance to Cd^2+^ compared with the other genotypes that had larger root systems. The tolerance of *Yidali* to Cd^2+^ stress which was demonstrated by the greatest tolerance indexes for RV, RSA, RPA, NRF and NRT could largely be attributed to the diminutive nature of its root system. The smaller root system of *Yidali* (especially fewer root tips and smaller surface area), probably limited the amount of Cd^2+^ absorbed by the roots. Liu, Probst & Liao (2005) attributed the lower Cd^2+^ contents in the roots of *S. alfedii* to the smaller size of root system (smaller root length, surface area and volume). Huang et al. (2015) also indicated that pepper cultivar with shorter roots, fewer root tips and smaller surface area had lower capacity for Cd^2+^ uptake. Moreover, the tolerance of plants to Cd^2+^ stress has also been assessed based on the content of H_2_O_2_ produced when the plants were exposed to the stress. Cd^2+^ toxicity results in increased production of reactive oxygen species such as H_2_O_2_ and superoxide anion which causes oxidative stress (Tripathi et al., 2017). Zhang et al. (2009) indicated that *Vicia sativa* which had lower content of H_2_O_2_ was more tolerant to Cd^2+^ than *Phaseolus aureus* which had higher content of H_2_O_2_ when both plants were exposed to Cd^2+^ treatment. Increase in the accumulation of H_2_O_2_ in lettuce plants under Cd^2+^ stress was earlier reported (Wang et al., 2019). Our experiment also showed that Cd^2+^ toxicity increased the H_2_O_2_ contents in the roots of the four lettuce genotypes. However, the increase in H_2_O_2_ was least in *Yidali*, and this also gives an indication of the tolerance of *Yidali* to Cd^2+^ stress compared with the other genotypes. These results, therefore, suggest that lettuce genotypes with smaller root systems could be more tolerant to Cd^2+^ stress compared with genotypes that possess larger root systems.

Research studies have shown that almost all hormones have the capacity to act as components of Cd^2+^-stress signaling in plants. The content of cytokinin (Gangwar et al., 2014), abscisic acid (Kim et al., 2014), gibberellin (Zhu et al., 2012), brassinosteroid (Villiers et al., 2011) and jasmonic acid (Zaid & Mohammad, 2018; Noriega et al., 2012) in plants have all been found to change in response to various stresses. Cd^2+^ stress increases the levels of ABA (Kim et al., 2014; Stroinski et al., 2010; Fediuc, Lips & Erdei, 2005) and GA (Zhu et al., 2012) in plants. However, the levels of IAA (Gangwar et al., 2014) and cytokinin (Veselov et al., 2003) decreased in plants under Cd^2+^ stress. In our study, the effect of Cd^2+^ on the contents of the hormones in the roots depended on the genotype. Under Cd^2+^ stress, ABA increased in *Anyan* and *Lüsu*, decreased in *Lümeng* and remained unaffected in the *Yidali* genotype. Moreover, under Cd^2+^ stress, the contents of cytokinin increased in *Anyan* and *Lüsu* but it decreased in *Yidali* and *Lümeng*. The variations in the hormonal responses to Cd^2+^ among the four genotypes could be attributed to differences in their sensitivity to Cd^2+^ stress. Different lettuce cultivars were found to have exhibited different levels of sensitivity to heavy metals (Priac, Badot & Crini, 2017). Hsu & Kao (2003) also found that the contents of ABA increased in the roots and leaves of Cd^2+^-tolerant rice cultivar but not in the Cd^2+^-sensitive cultivar.

Under the control condition, ABA and IAA contents in roots correlated positively with most of the root indexes whiles GA_3_ and cytokinin correlated negatively with TRL and RPA respectively. ABA generally acts as growth inhibitor under stress free conditions (Takezawa, Komatsu & Sakata, 2011). In our study, ABA correlated positively with most of the root morphological indexes under Cd^2+^ stress. This suggests that ABA can play a positive role in promoting the tolerance of lettuce plants to Cd^2+^ stress. This observation could be attributed to the low concentrations of ABA in the roots of the genotypes tested in the current experiment. Barlow & Pilet (1984) reported that low concentrations of ABA increased root elongation, cell division and DNA synthesis whiles high concentration decreased root growth in maize. The promotion of root growth due to auxin was also demonstrated when root morphological parameters increased upon the application of 5–20 ppm IAA to grafted cucumber seedlings (Balliu & Sallaku, 2017). Although both cytokinin (Mok, 1994) and GA_3_ (De Souza & MacAdam, 2001) promote growth through cell division, cell expansion and cell elongation in this experiment. These hormones correlated negatively with TRL and RPA, respectively. This suggests that increasing cytokinin and GA_3_ contents in the roots of lettuce under Cd^2+^ free condition may decrease root growth. However, under Cd^2+^ stress, GA_3_ andIAA contents of roots correlated negatively with some of the root indexes but ABA correlated negatively with most of the root indexes compared with GA_3_ andIAA. Although under the control condition, both GA_3_ and IAA correlated positively with root morphological indexes, both hormones correlated negatively with the root indexes under Cd^2+^ stress. This suggests that the interaction effect of these hormones may not influence root growth in lettuce but could enhance the tolerance of the plants to Cd^2+^ stress. The positive correlation between ABA and GA_3_ and also between ABA and IAA under Cd^2+^ stress is indicative of the importance of ABA in alleviating Cd^2+^ stress in lettuce plants. ABA is reported to play significant role in plants response to various stresses (Kiba et al., 2011). In plants under drought stress, ABA alleviated the stress by inducing stomata closure, leaf folding and osmotic adjustment (Lipiec et al., 2013). Under stress free conditions, Cytokinin generally suppresses growth and development of roots (Werner et al., 2003) and it is usually antagonistic with IAA in roots (Nordström et al., 2004). However, in this experiment, only cytokinin correlated positively with most of the root indexes under Cd^2+^ stress. The positive correlation between cytokinin and most of the root morphological indexes suggests that increased level of cytokinin may be required to support the survival of lettuce plants under Cd^2+^ stress. Vitti et al. (2013) found that cytokinin increased root growth of *A. thaliana* seedlings and improved their tolerance to Cd^2+^ stress.

The antioxidant enzymes activities and proline response of plants to heavy metal stress depends on species, cultivar, Cd^2+^ concentration and duration of exposure. The enzymatic defense system in plants includes SOD, CAT, and ascorbate peroxidase (Foroozesh et al., 2012). The SOD is a major scavenger which catalyzes the conversion of superoxide to H_2_O_2_ and molecular oxygen under stress conditions (Allen, 1995). The H_2_O_2_ which is also toxic to plant cells is detoxified by CAT and/or POD, which converts it to water and oxygen (Zhu et al., 2004). Proline is also among the components of the non-specific defense systems in plants. It alleviates metal toxicity by acting as a metal chelator and also as a protein stabilizer (Sharma & Dubey, 2005). In the current experiment, antioxidant enzymes activities and proline response to Cd^2+^ stress in the roots of the four lettuce genotypes were similar. Although the SOD activity of the genotypes decreased at seven days after exposure to Cd^2+^ stress, the CAT and POD activities, as well as the contents of proline increased. The decrease in SOD activity observed in all the genotypes at seven days after the Cd^2+^ treatment could be due to fact that the SOD which is the first enzyme involved in the antioxidant enzyme defense system might have completed its function within few days after the Cd^2+^ treatment, which resulted in the increased H_2_O_2_ contents. The increase in CAT and POD activities was to enable the plants further detoxify the H_2_O_2_ which is also toxic to plant cells. Decreased SOD activity due to Cd^2+^ stress was reported in lettuce (Xu et al., 2014), *A. thaliana* (Abozeid et al., 2017) and in *P. sativum* L. (Rodriguez-Serrano et al., 2006). The CAT and POD activities and the contents of proline in the roots of the lettuce genotypes exposed to Cd^2+^ increased to counteract the Cd^2+^-induced oxidative stress (increased H_2_O_2_ contents) in the plants. In a previous experiment, POD, SOD and CAT activities and the content of proline in oilseed rape plants exposed to Cd^2+^ stress increased (Yan et al., 2015). In other experiments, the contents of proline in different lettuce varieties increased (Jibril et al., 2017) and the POD activity in pea seedlings also increased (Agrawal & Mishra, 2007) increased when these plants were exposed to Cd^2+^ stress.

## Conclusions

In this study, the root tolerance and biochemical response of four lettuce genotypes to100 µM CdCl_2_ (Cd^2+^) treatment were determined. The results showed that Cd^2+^ stress decreased the sizes of the root systems of the four lettuce genotypes evaluated. However, the *Yidali* genotype, which possessed the smallest root system, exhibited the greatest tolerance to Cd^2+^ stress with the by exhibiting the greatest tolerance index scores for root volume, surface area, projected area and numbers of root forks and root tips. Cd^2+^ stress also caused increases in H_2_O_2_ contents in the roots of the genotypes but the increase was least in the *Yidali* genotype which showed more root tolerance to Cd^2+^stress. The effect of Cd^2+^ stress on ABA, IAA, GA_3_ and cytokinin was genotype dependent. Under Cd^2+^ stress, the correlation between ABA and IAA, GA_3_ and cytokinin were positive. The antioxidant enzyme activities and proline response to Cd^2+^ stress in the roots of the four genotypes were similar. The SOD activity decreased whiles the CAT and POD activities, as well as the contents of proline, increased under the stress condition. Greater root tolerance indexes and lower H_2_O_2_ content were found in the *Yidali* genotype under the stress condition, suggesting that lettuce genotypes with smaller root system could be more tolerant to Cd^2+^ stress. However, further research is needed to ascertain the details of the molecular and genetic mechanisms of Cd^2+^ tolerance in lettuce genotypes with relatively smaller root systems.

##  Supplemental Information

10.7717/peerj.7530/supp-1 Data S1 Lettuce genotypes used for the study

## Additional Information and Declarations

### Data Availability

The following information was supplied regarding data availability: The raw data are available in the Supplemental File. The raw data shows measurements of root morphological indexes, contents of root hormones and proline and the antioxidant enzymes activities in the roots of four lettuce genotypes under cadmium and control conditions.
